# Supplementary material for: Partially overlapping spatial environments trigger reinstatement in hippocampus and schema representations in prefrontal cortex
Source: Nat Commun. 2021 Oct 28;12:6231. doi: 10.1038/s41467-021-26560-w (PMC8553856; doi:10.1038/s41467-021-26560-w)
Supplement: Supplementary file 1 — Supplementary Information [file 41467_2021_26560_MOESM1_ESM.pdf]

Supplementary Information

Partially overlapping spatial environments trigger reinstatement in hippocampus and schema representations in prefrontal cortex

Zheng, L., Gao, Z.Y., McAvan, A., Isham E., and Ekstrom A.D*.

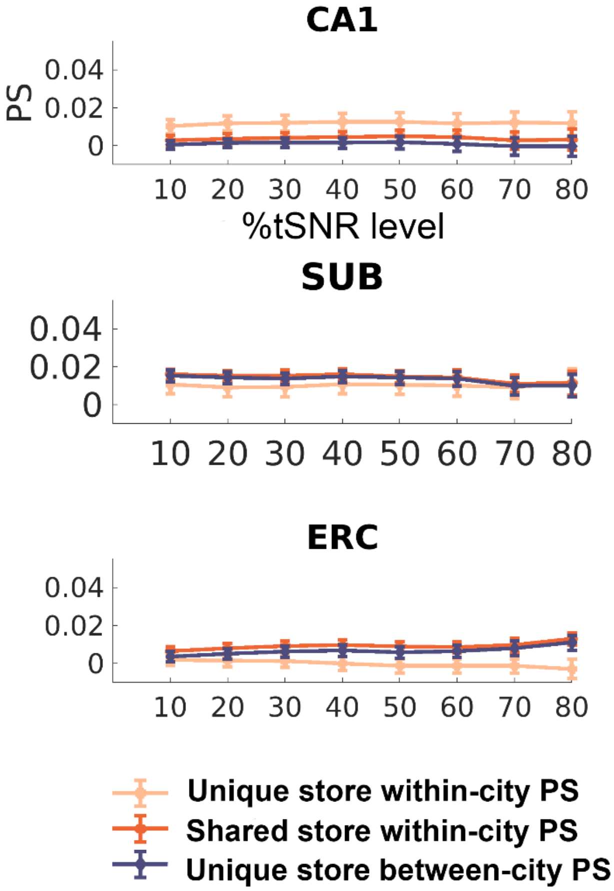

Supplementary Figure 1. Multivariate pattern similarity analysis (MPS) of holistic retrieval in hippocampus at each tSNR level. The neural representations of the three conditions (unique store within-city PS / shared store within-city PS / unique store between-city PS) at each tSNR level in each ROI. From the left to right, the number of voxels is decreasing by removing more and more spurious voxels. Notes: Data were presented as mean ± SEM and all data reflect n = 27 independent participants. Source data are provided in the Source Data file.

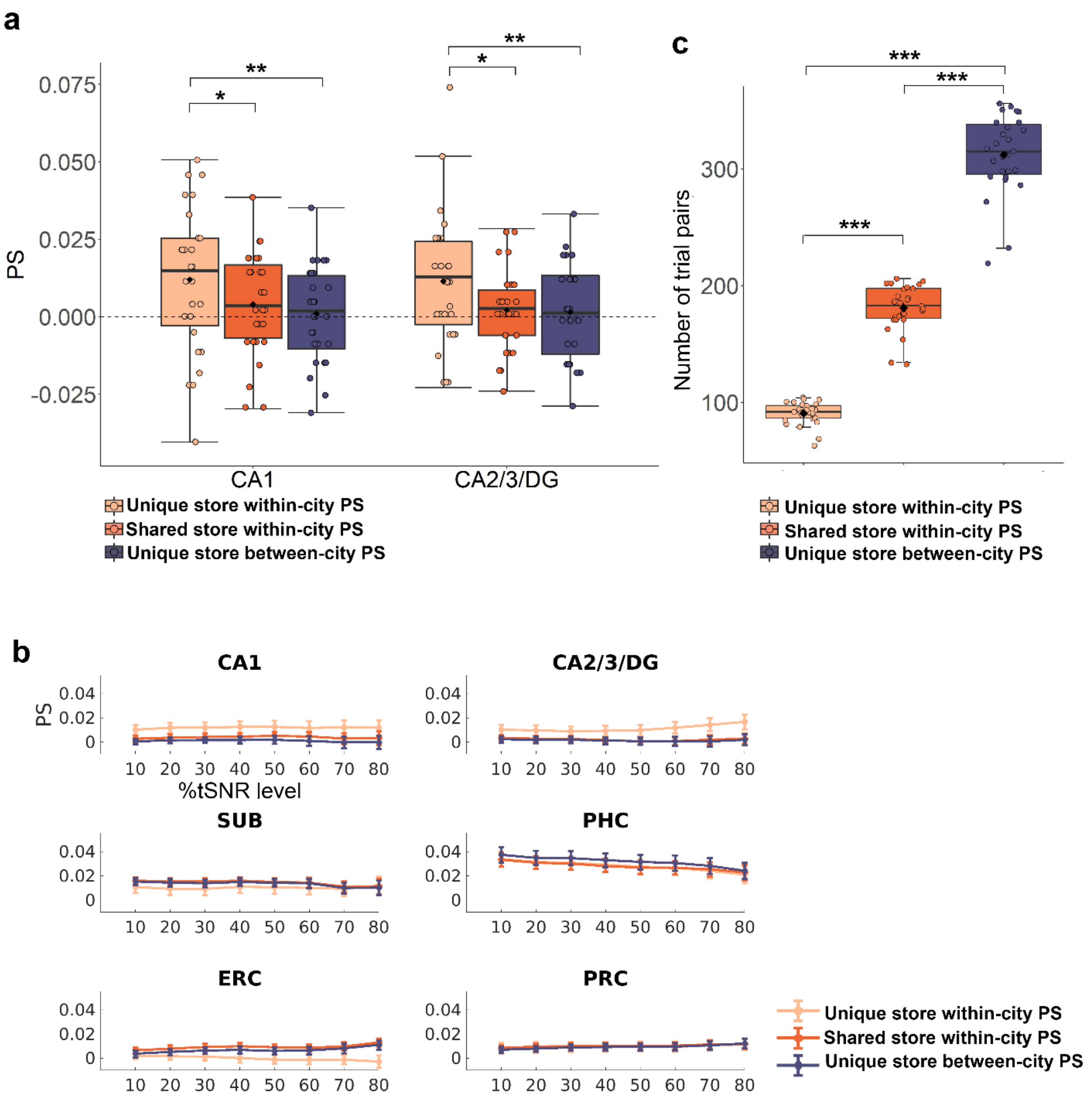

Supplementary Figure 2. Multivariate pattern similarity analysis (MPS) of holistic retrieval in hippocampus after matching trial pairs of each condition. a Holistic and differentiated representations in hippocampus during retrieval (averaged across 8 tSNRs). Consistent with the third hypothesized outcome (Fig.3), the neural representations in CA1 and CA2/3/DG were holistic (CA1: $p = 0.0057$, CA2/3/DG: $p = 0.0057$, two-tailed paired-sample t-test with FDR correction) and differentiated from other city representations (CA1: $p = 0.021$, CA2/3/DG: $p = 0.022$, two-tailed paired-sample t-test with FDR correction). **b** The neural representations of three conditions (unique store within-city PS / shared store within-city PS / unique store between-city PS) at each tSNR level in each ROI. From the left to right, the number of voxels is decreasing by removing more and more spurious voxels. **c** The number of trial pairs that went into each condition in the MPS analysis (*Ps* < 0.001, two-tailed paired-sample

t-test with FDR correction). Data were presented as mean ± SEM. Notes: Boxplots are centered on the median, boxes extend to first and third quartiles, whiskers extend to 1.5 times the interquartile range or minima/maxima in the absence of outliers. Each unfilled dot represents data from individual subjects. Each black solid diamond represents the mean of the group. All data reflect n = 27 independent participants. SUB: subiculum, PRC: perirhinal cortex, ERC: entorhinal cortex, PHC: parahippocampus cortex. PS: pattern similarity. *p < 0.05, **p < 0.01, ***p < 0.001. Source data are provided in the Source Data file.

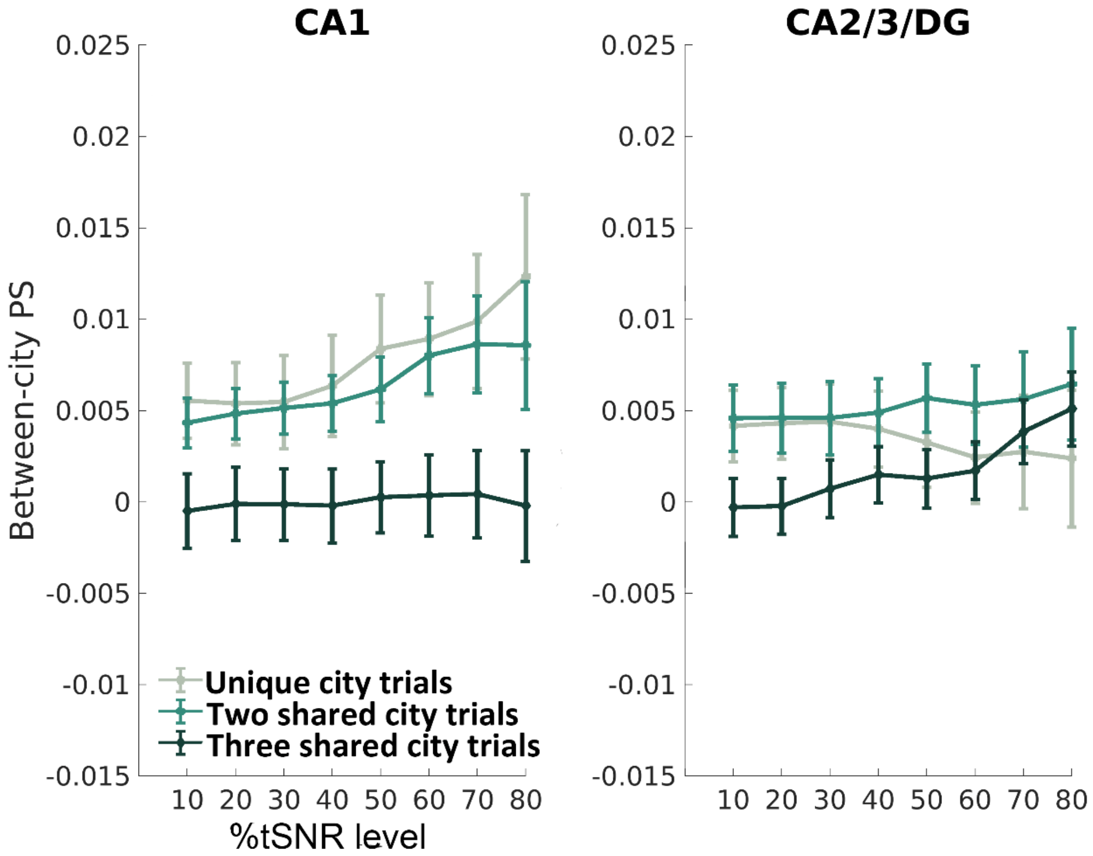

Supplementary Figure 3. Between city PS for unique city trials, two shared city trials, three shared city trials) at each tSNR level of CA1 and CA2/3/DG. From the left to right, the number of voxels is decreasing by removing more and more spurious voxels. Notes: PS: pattern similarity. Data were presented as mean ± SEM. All data reflect n = 27 independent participants. Source data are provided in the Source Data file.

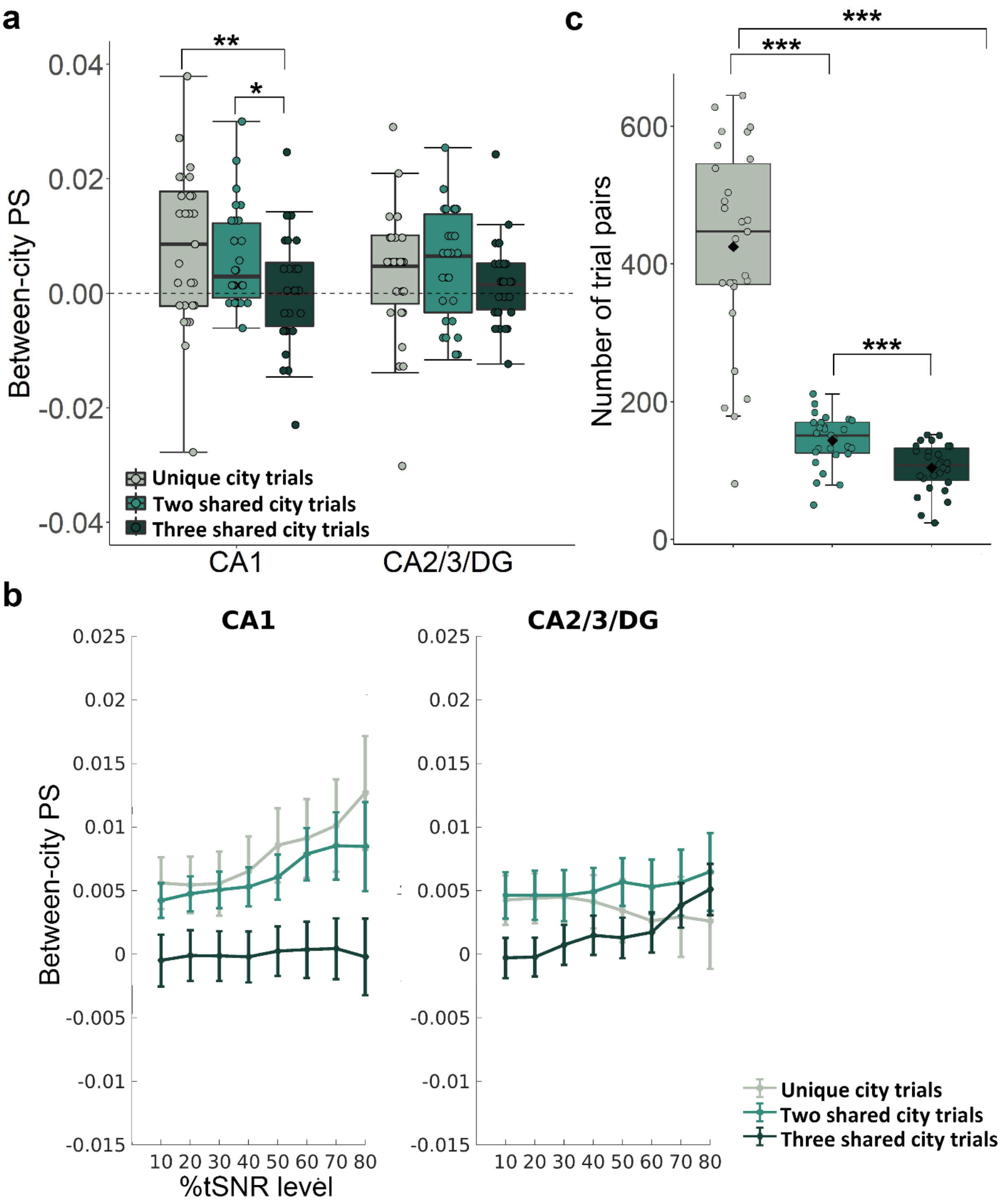

Supplementary Figure 4. Multivariate pattern similarity analysis (MPS) of differentiated representations for shared city trials in hippocampus after matching trial pairs of each condition. a The neural representations for shared-city

trials showed a repulsion effect in CA1 (consistent with the third hypothesized outcome, $p = 0.0036$, two-tailed paired-sample t-test with FDR correction) and a pattern separation effect in CA2/3/DG (consistent with the third hypothesized outcome, averaged across 8 tSNRs). The two city shared trials did not show lower between-city PS than unique city trials ($p = 0.610$, two-tailed paired sample t-test with FDR correction) but significant higher between-city PS than three city shared trials ($p = 0.011$, two-tailed paired-sample t-test with FDR correction). **b** The neural representations for between-city PS for unique city trials, two shared city trials, and three shared city trials at each tSNR level for CA1 and CA2/3/DG. From the left to right, the number of voxels is decreasing by removing more and more spurious voxels. **c** The number of trial pairs that went into each condition in the MPS analysis (*Ps* < 0.001, two-tailed paired-sample t-test with FDR correction). Data were presented as mean ± SEM. Notes: Boxplots are centered on the median, boxes extend to first and third quartiles, whiskers extend to 1.5 times the interquartile range or minima/maxima in the absence of outliers. Each unfilled dot represents data from an individual subject. The solid black solid diamonds represent the mean of the group. All data reflect n = 27 independent participants. PS: pattern similarity. *$p < 0.05$, **$p < 0.01$, ***$p < 0.001$. Source data are provided in the Source Data file.

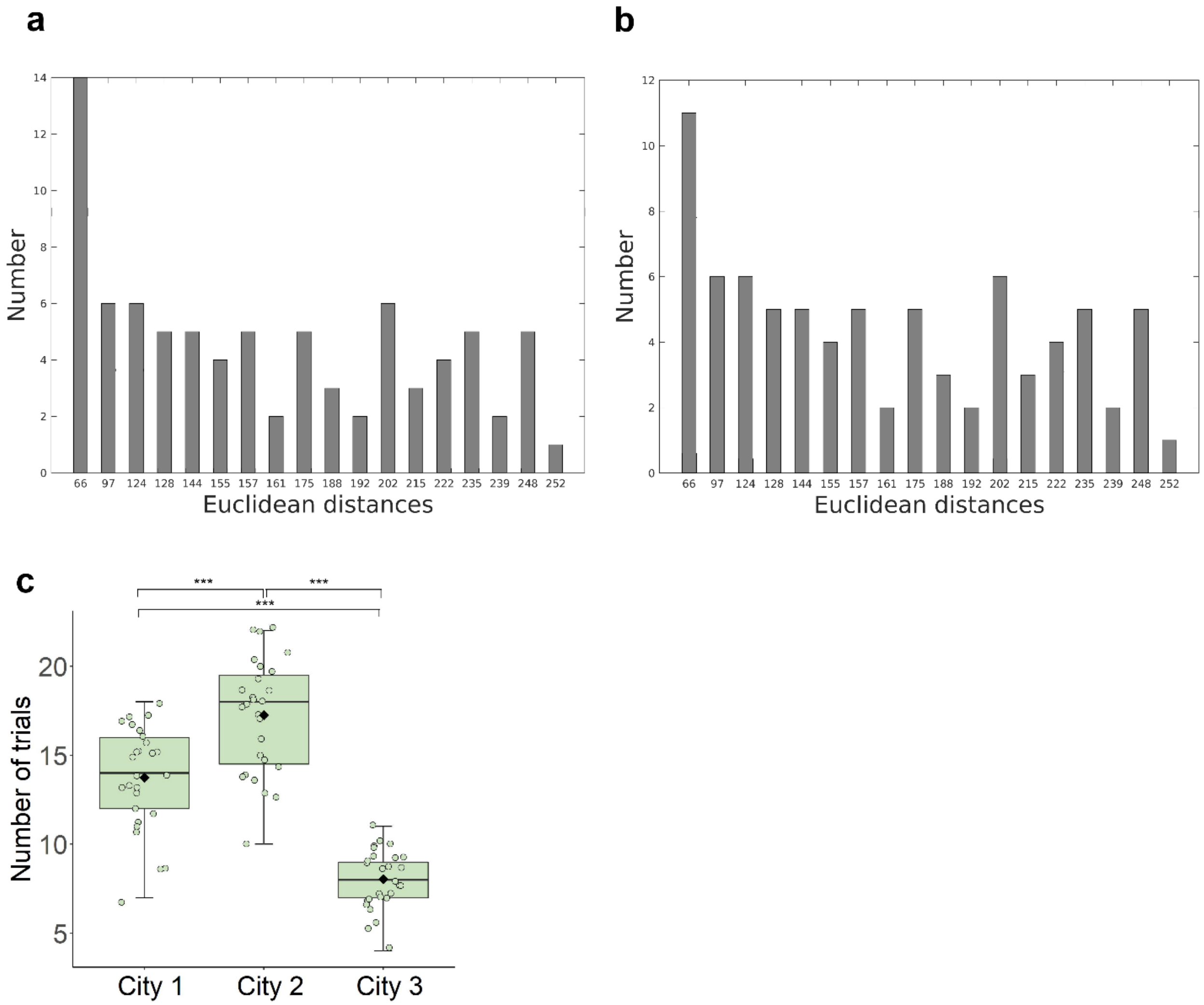

Supplementary Figure 5. The distributions of Euclidean distances and control analysis of schematic spatial layout representation in medial PFC. a The group level distribution of Euclidean distances across trials was not uniformity (p = 0.016), with shorter distances overrepresented. **b** After randomly removing 3 trials of the shortest distance, the distribution was significantly uniform (p = 0.08). **c** The count of shared trials across cities in each leave-one-city-out classification iteration. For example, when City 1 and City 2 were used as the training set, and City 3 as the test set, we could calculate how many trials of City 1 and 2 were shared with City 3 (*Ps* < 0.001, two-tailed paired-sample t-test with FDR correction). Notes: Boxplots are centered on the median, boxes extend to first and third quartiles, whiskers extend to 1.5 times the interquartile range or minima/maxima in the absence of outliers Each unfilled dot represents data from an individual subject. Solid black diamonds represent the mean of the group. All data reflect n = 27 independent participants. ***p < 0.001. Source data are provided in the Source Data file.

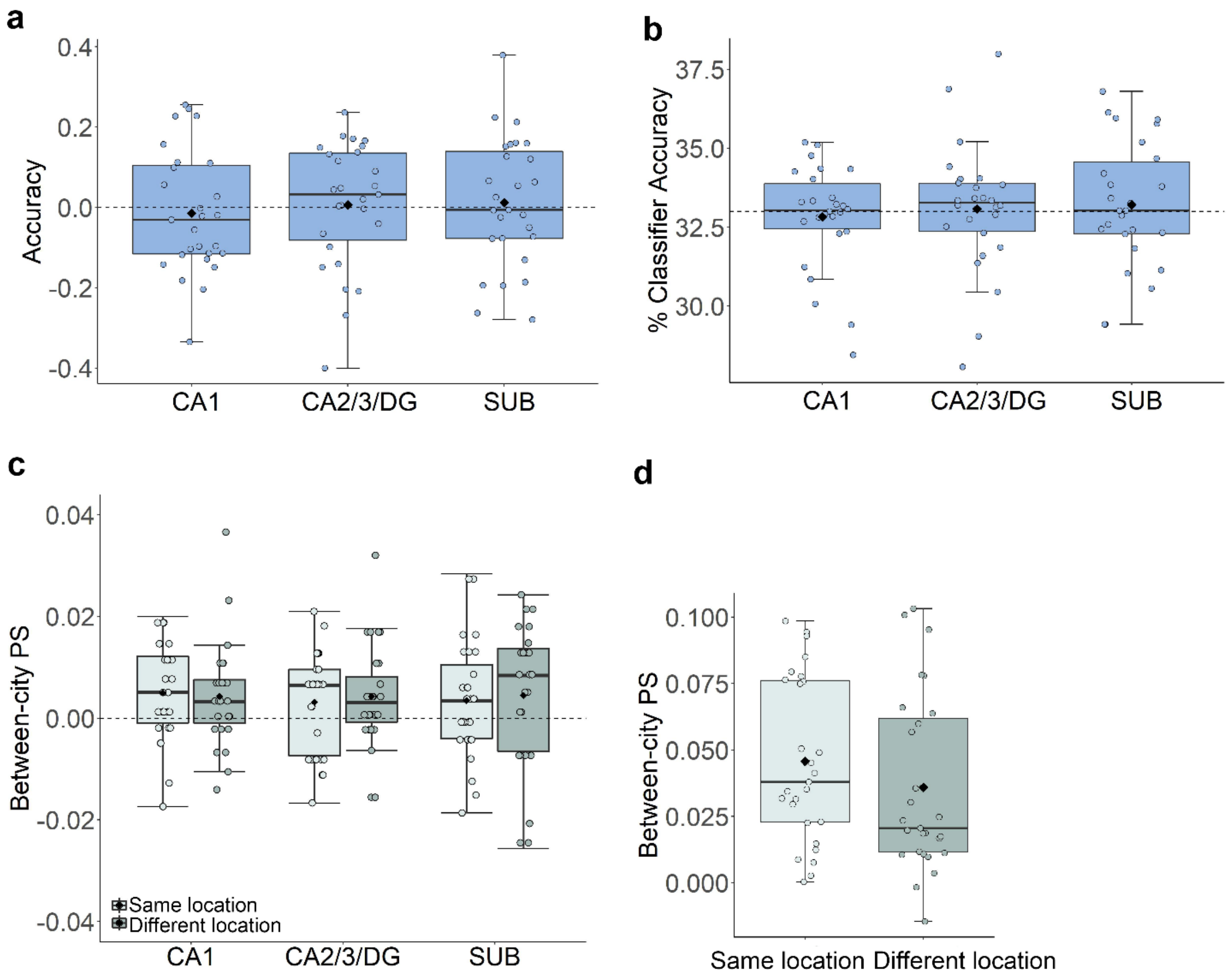

Supplementary Figure 6. There was no schematic spatial layout representation in hippocampus. a No ROIs within the hippocampus showed above chance level classification for spatial distance estimates. Data reflect n = 27 independent participants. **b** No ROIs within the hippocampus showed above chance level classification for temporal interval estimates. Note: Data reflect n = 26 independent participants. One subject was additionally excluded from this analysis because of not enough trials in one of the three time interval conditions. **c** No hippocampal ROIs showed higher between-city PS for same locations than different locations. Data reflect n = 27 independent participants. **d** The averaged between-city PS for same locations and different locations from the cluster of media frontal pole in the searchlight MPS analysis (note: this figure is only for showing the PS of each condition and no t-test was applied between conditions to avoid a double-dipping problem). Data reflect n = 27 independent participants. Notes: Boxplots are centered on the median, boxes extend to first and third quartiles, whiskers extend to 1.5 times the interquartile range or minima/maxima in the absence of outliers. Each unfilled dot represents data from an individual subject. Each black solid diamond represents the mean of the group. PS: pattern similarity, SUB: subiculum. Source data are provided in the Source Data file.

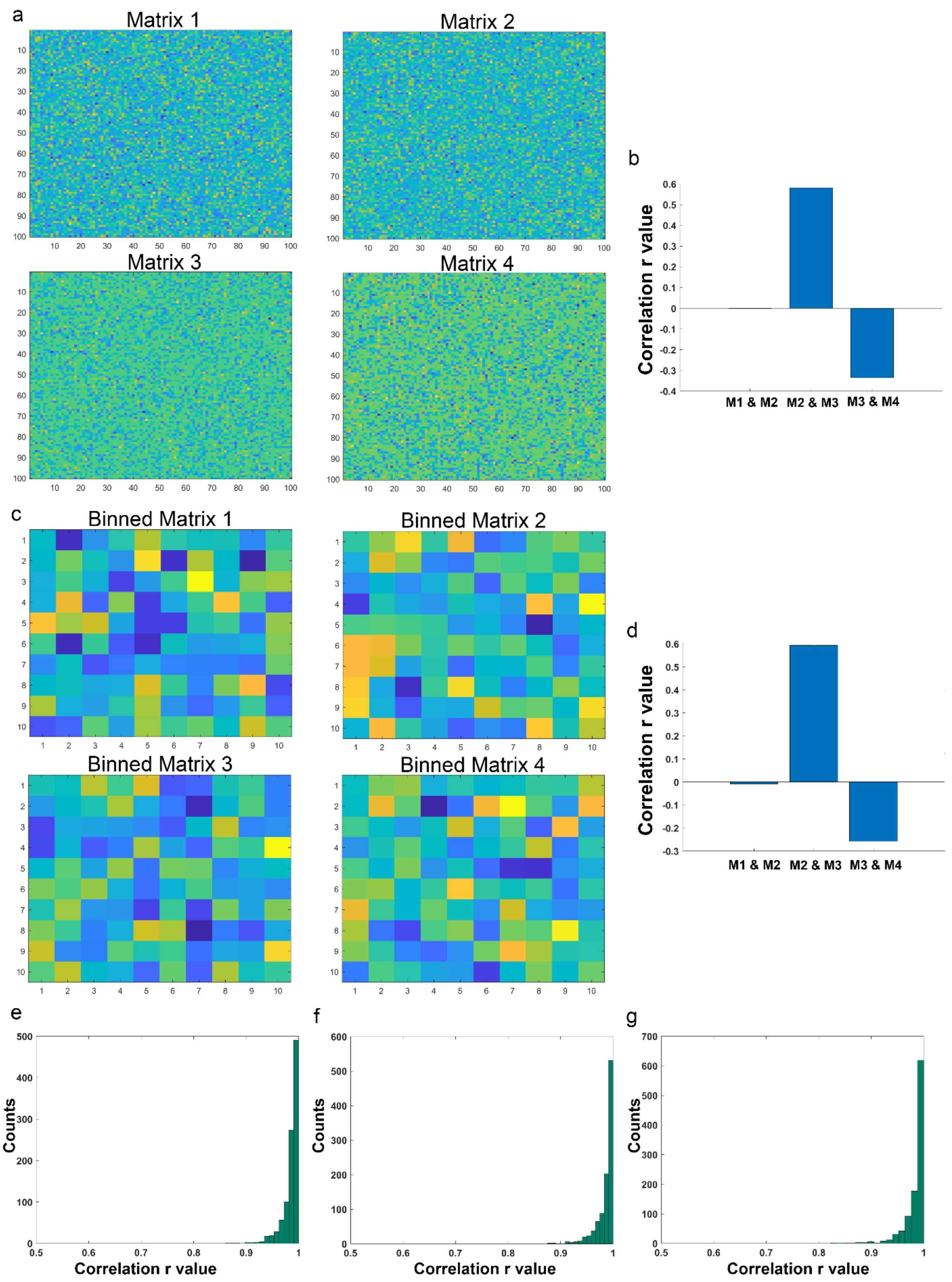

a
Matrix 1
Matrix 2
Matrix 3
Matrix 4
b
Correlation r value
M1 & M2
M2 & M3
M3 & M4
c
Binned Matrix 1
Binned Matrix 2
Binned Matrix 3
Binned Matrix 4
d
Correlation r value
M1 & M2
M2 & M3
M3 & M4
e
f
g
Counts
Correlation r value

Supplementary Figure 7. The simulation results. a Each matrix represents a collection of randomly distributed hippocampal neurons active in some form during exploration of four different environments. **b** The correlation between matrix 1&2, matrix 2&3, and matrix 3&4. **c** Binned matrices (with voxels 1/10th the original resolution) generated from original matrices in a. **d** The correlation between binned matrix 1&2, binned matrix 2&3, and binned matrix 3&4. **e** The correlation between the original and binned matrix correlations in 1000 times simulation with new random values and matrices. **f** The correlation between the original and binned matrix correlations in 1000 times simulation when using zeros to create matrix 3 and 4. **g** The correlation between the original and binned matrix correlations in 1000 times simulation when using random numbers to create matrix 3 and 4. Note: M1: Matrix 1, M2: Matrix 2, M3: Matrix 3, M4: Matrix4.

Supplementary Table 1. Unique store within-city PS for CA1 and CA2/3/DG (compared with zero, two-tailed one-sample test. No correction for multiple comparisons was applied)

Task	Region	Mean	SD	t	p
Space and Time	CA1	0.012	0.023	2.661	0.013
	CA2/3/DG	0.012	0.022	2.707	0.012
Space	CA1	0.019	0.022	4.637	0.001
	CA2/3/DG	0.012	0.030	2.118	0.044
Time	CA1	0.007	0.032	1.173	0.251
	CA2/3/DG	0.010	0.030	1.828	0.078

Supplementary Table 2. Shared store within-city PS for CA1 and CA2/3/DG (compared with zero, two-tailed one-sample test. No correction for multiple comparisons was applied)

Task	Region	Mean	SD	t	p
Space and Time	CA1	0.004	0.017	1.220	0.234
	CA2/3/DG	0.002	0.013	0.852	0.402
Space	CA1	0.006	0.024	1.326	0.196
	CA2/3/DG	0.006	0.015	1.894	0.069
Time	CA1	0.003	0.024	0.625	0.537
	CA2/3/DG	0.0001	0.018	0.031	0.975

Supplementary Table 3. Unique store between-city PS for CA1 and CA2/3/DG (compared with zero, two-tailed one-sample test. No correction for multiple comparisons was applied)

Task	Region	Mean	SD	t	p
Space and Time	CA1	0.001	0.016	0.355	0.725

	CA2/3/DG	0.002	0.016	0.486	0.631
Space	CA1	0.002	0.022	0.509	0.615
	CA2/3/DG	0.003	0.019	0.862	0.396
Time	CA1	0.0005	0.025	0.108	0.915
	CA2/3/DG	0.0003	0.020	0.082	0.935

Supplementary Note 1: behavioral results for spatial and temporal retrieval.
We conducted a 2 (task: spatial, temporal) × 3 (city: City 1, City 2, City 3) repeated-measures ANOVA on memory accuracy. The analysis showed no main effect of task ($F(1,26) = 2.624$, $p = 0.117$, $\eta^2_p = 0.092$) or city ($F(2,52) = 1.024$, $p = 0.366$, $\eta^2_p = 0.038$) and no interaction effect between task and city ($F(2,52) = 1.822$, $p = 0.172$). Post hoc comparisons of memory accuracy revealed no significant differences across the three cities ($t(26) < 1.359$, *Ps* > 0.540, Cohen's d < 0.262). The Bayes factor calculated for the memory accuracy suggested that the result was more likely obtained under the null hypothesis that there was no difference between two tasks ($BF_{10} = 0.647$, with anecdotal evidence) and across three cities ($B_{10} < 0.484$, with anecdotal evidence).

Similarly, the same two-way repeated ANOVA on reaction time (RT) also revealed no significant main effect of city ($F(2,52) = 0.252$, $p = 0.778$, $\eta^2_p = 0.010$). However, there was a significant main effect of task ($F(1,26) = 219.546$, $p < 0.001$, $\eta^2_p = 0.894$). The Bayes factor calculated on RT for the space versus temporal tasks suggested that the result was obtained under the alternative hypothesis (with strong evidence) that RT was significantly slower ($BF_{10} = 137200000000$) in the spatial task (mean = 4.316 s, SD = 0.601) than in temporal task (mean = 2.705 s, SD = 0.757). Meanwhile, the Bayes factor calculated for RT for both tasks suggested that result was more likely obtained under the null hypothesis that there was no difference across the three cities ($F_{10} < 0.259$, with moderate evidence).

To rule out the possibility that the repulsion effect was driven by differences in behavioral performance in three conditions (trial type: unique city trials, two shared city trials, and three shared city trials), we conducted a one-way repeated ANOVA (trial type: unique city trials, two shared city trials and three shared city trials) on RT. This analysis showed no significant main effect of trial type ($F(1.388, 36.091) = 1.399$, $p = 0.256$, $\eta^2_p = 0.051$, Greenhouse-Geisser corrected). Post hoc comparisons of memory accuracy revealed no significant differences between the three trial types ($t(26) < 1.596$, *Ps* > 0.349, Cohen's d < 0.307). The Bayes factor calculated for RT suggests that result was more likely obtained under the null hypothesis that there was no difference across three trial types (unique city trials vs. three shared city trials: $B_{10} = 0.215$, with moderate evidence; unique city trials vs. two shared city trials: $B_{10} = 1.216$, with anecdotal

evidence; two shared city trials vs. three shared city trials: B_{10} = 0.440, with anecdotal evidence).

We calculated the behavioral performance for the localizer (vowel counting) task and compared to the memory retrieval task. The results showed that RT was significantly longer for the vowel counting task (mean = 5.823 s, SD = 0.824) than spatial retrieval task (mean = 4.317 s, SD = 0.601; $t(26) = 11.008$, $p < 0.001$, CI = [1.507 ± 0.282], Cohen's d = 2.118) and temporal retrieval (mean = 2.705 s, SD = 0.757; $t(26) = 18.776$, $p < 0.001$,CI = [3.119 ± 0.341], Cohen's d = 3.613). Accuracy for the vowel counting task (mean = 0.724, SD = 0.208) was significantly worse than the spatial retrieval task ($t(26) = -3.461$, $p = 0.002$, CI = [-0.126 ± 0.075], Cohen's d = -0.666) and the temporal retrieval task ($t(26) = -3.906$, $p < 0.001$, CI = [-0.161 ± 0.085], Cohen's d = -0.752). This suggested that the vowel counting task was more demanding than the memory retrieval task and indicated that participants were indeed paying close attention to the task.

Supplementary Note 2: holistic representations in hippocampus (space/time task). Focusing on the two hippocampal subfields (i.e., CA1, CA2/3/DG) which support holistic retrieval, we further tested whether hippocampal representations were holistic when the spatial or temporal task was considered separately. First, we examined this hypothesis in spatial task. The results revealed that the within-city PS for unique stores was significantly higher than between-city PS for unique stores in CA1 ($t(26) = 3.559$, $p = 0.0015$, CI = [0.017 ± 0.01], Cohen's d = 0.685, two-tailed) and but not significant in CA2/3/DG ($t(26) = 1.705$, $p = 0.100$, CI= [0.009 ± 0.01], Cohen's d = 0.328, two-tailed). Similarly, we examined this hypothesis in temporal task. The results revealed that the within-city PS for unique stores was significantly higher than between-city PS for unique stores in CA2/3/DG ($t(26) = 2.391$, $p = 0.024$, CI = [0.01 ± 0.009], Cohen's d = 0.460, two-tailed) but not significant in CA1 ($t(26) = 1.617$, $p = 0.118$, CI = [0.007 ± 0.008], Cohen's d = 0.311, two-tailed).

Supplementary Note 3: addressing possible confounds in MPS analysis. One potential issue in terms of the differences between unique store within-city PS and shared store within-city PS could be driven by the differences of activation levels for different stores. Therefore, we tested six ROIs (CA1, CA2/3/DG, SUB, ERC, PRC, PHC) and 6 stores in a repeated measures ANOVA based on the mean activation in localizer task. This allowed us to test whether the MPS differences could be explained based on differences in univariate activation levels. This analysis did not reveal any significant main effect of store or interactions between ROI and store (main effect of store: $F(5, 130) = 1.008$, $p = 0.416$, $\eta^2_p = 0.037$; interaction effect: $F(11.558,300.515) = 1.217$, $p = 0.272$, $\eta^2_p = 0.045$, Greenhouse-Geisser corrected). Furthermore, depending

on the types of stores (i.e., unique stores and shared stores), we also specifically tested whether the two types of stores showed different activation levels by performing an ROI by store type repeated measures ANOVA. This revealed no significant effects (main effect of store type: $F(1,26) = 0.761$, $p = 0.391$, $\eta^2_p = 0.028$; interaction effect: $F(5,130) = 0.937$, $p = 0.459$, $\eta^2_p = 0.035$). These findings suggest that the differences between unique store within-city PS and shared store PS could not be accounted by the store differences in univariate activation levels.

Another potential issue that may contribute to the holistic retrieval is the semantic association of stores. We tested whether the within-city between stores semantic associations was significantly higher than the between-city semantic associations. We obtained the distributed representations of meanings of each store by using word2vec neural network[1]. Within-city semantic association was defined as the cosine similarity between each store within one city, while the between-city semantic association was defined as the cosine similarity between each store between cities. This analysis showed no significant difference between within-city and between-city semantic association ($p = 0.226$). These results suggest that the differences between unique store within-city PS and unique store between-city PS could not be accounted by the semantic association between stores.

In addition, compared to unique-store trials, three shared city trials were presented in all three cities. Could the repulsion effects we found in CA1 be driven by repetition suppression? In order to exclude this possibility, we conducted a 2 (task: spatial, temporal) × 3 (city: City 1, City 2, City 3) repeated measures ANOVA on activation in CA1. The analysis showed no main effect in task ($F(1,26) = 0.068$, $p = 0.796$, $\eta^2_p = 0.003$) or city ($F(2,52) = 0.237$, $p = 0.790$, $\eta^2_p = 0.009$) and no interaction effect between task and city ($F(1.531,39.811) = 2.230$, $p = 0.118$, $\eta^2_p = 0.079$). Post hoc comparisons of activation in CA1 revealed no significant differences across three cities (*Ps* > 0.539).

It is important to examine whether the findings we found exist outside the MTL. To address this concern, first, we tested whether the representation of other brain regions outside MTL are also holistic by using both ROI-based and searchlight-based MPS. In the ROI-based MPS analysis, we created ROIs using meta-analysis in Neurosynth (https://neurosynth.org/) with the key term “memory retrieval”. Based on the Neurosynth associative tests, a threshold Z-score > 3.1 was applied to create clusters. There were 9 ROIs, including middle frontal gyrus, inferior frontal gyrus, medial frontal cortex, frontal pole, lateral occipital cortex, precuneus, paracingulate, middle temporal gyrus, temporal pole. However, the results indicated that no ROIs showed significantly higher within-city PS for unique stores than between-city PS for unique stores (*Ps* > 0.121). Similarly, no cluster was found in a searchlight-based MPS across the whole brain. Therefore, all further analysis were focused on hippocampal subfields. Overall,

our findings related to holistic representation and repulsion effects appear specific to the hippocampus, at least within our data.

Finally, in all the MPS analysis, we randomly resampled a subset of trial pairs from each condition so that the difference between conditions would be calculated using the same number of trial pairs (see below). This helped to rule out the possibility that our results were not driven by unequal trial pairs between conditions[2].

Supplementary Note 4 (related to supplementary Figure 2): representations in hippocampus are holistic after matching the trial pairs of each condition. Because the trial pairs of each condition (i.e., unique store within-city PS / shared store within-city PS / unique store between-city PS) were unequal (Supplementary Figure 2c), we conducted a control analysis to match the trial pairs of each condition by randomly resampling the pairs of the condition which has more pairs with the smaller number of pairs 5000 times. We conducted a three-way repeated-measures ANOVA, with the factors of 6 ROIs of MTL, 8 t-stat thresholds (tSNR) levels, and three conditions (unique store within-city PS / shared store within-city PS / unique store between-city PS) as within-subject variables. The results revealed a significant ROI-by-condition interaction ($F(5.522, 143.580) = 4.954$, $p < 0.001$, $\eta^2_p = 0.160$, Greenhouse-Geisser corrected). Because there was no significant interaction of tSNR by ROI by condition ($F(7.740,201.249) = 1.590$, $p = 0.132$, $\eta^2_p = 0.058$, Greenhouse-Geisser corrected), we averaged pattern similarity for each ROI with different tSNR levels for the following analyses (Supplementary Figure 2). Simple effects revealed that the within-city PS for unique stores was significantly higher than between-city PS for unique stores both in CA1 ($t(26) = 3.559$, $p = 0.0057$, CI = [0.011 ± 0.006], Cohen's d = 0.685, two-tailed, FDR corrected) and CA2/3/DG ($t(26) = 3.454$, $p = 0.0057$, CI = [0.010 ± 0.006], Cohen's d = 0.665, two-tailed, FDR corrected) whereas the within-city PS for shared stores was not significantly different from between-city PS for unique stores (CA1: $t(26) = 1.844$, $p = 0.077$, CI = [0.003 ± 0.003], Cohen's d = 0.355; CA2/3/DG: $t(26) = 0.411$, $p = 0.685$, CI = [0.001 ± 0.003], Cohen's d = 0.079, two-tailed, Supplementary Figure 2a). These findings suggest that spatial retrieval was holistic and specific to unique information of an environment (and not the shared information). Furthermore, within-city PS was higher for unique stores than that for stores shared across cities in CA1 ($t(26) = 3.135$, $p = 0.021$, CI = [0.008 ± 0.005], Cohen's d = 0.603, two-tailed, FDR corrected) and CA2/3/DG ($t(26) = 2.730$, $p = 0.022$, CI = [0.009 ± 0.007], Cohen's d = 0.526, two-tailed, FDR corrected), supporting the idea that such holistic representations were also differentiated from other city representations. This control analysis demonstrates that the number of trials was not a confounding factor in the analysis reported in the results section comparing within and between city PS for unique store retrieval.

Supplementary Note 5 (related to Supplementary Figure 4): Hippocampal neural codes for shared city trials involve differentiated and "repulsed" representations after matching the trial pairs for each condition. Given the trial pairs of each condition (i.e., unique city trials, two shared city trials, three shared city trials) were unequal (Supplementary Figure 4c), we conducted a control analysis to match the trial pairs of each condition by randomly resampling the unique city trial pairs with the smaller number of two/three shared city trial pairs. We repeated this procedure repeated 5000 times.

We conducted a two-way repeated measure ANOVA, with the factors of 8 tSNR levels and three conditions (between-city PS for unique city trials, between-city PS for two shared city trials and between-city PS for three shared city trials) as within-subjects variables in CA1 and CA2/3/DG separately. We found a significant main effect of conditions in CA1 ($F(1.642, 42.7) = 5.670$, $p = 0.010$, $\eta^2_p = 0.179$, Greenhouse-Geisser corrected), but not in CA2/3/DG ($F(1.503, 39.068) = 0.979$, $p = 0.363$, $\eta^2_p = 0.036$, Greenhouse-Geisser corrected). Because there was no significant interaction of tSNR by condition in the two ROIs (*Ps* > 0.187, Greenhouse-Geisser corrected), we then averaged pattern similarity for each ROI across different tSNR levels for the post-hoc paired t-test for three conditions.

We found that between-city PS for unique-city trials was significantly higher than three shared city trials ($t(26) = 3.487$, $p = 0.0036$, CI = [0.008 ± 0.005], Cohen's d = 0.671, two-tailed, FDR corrected) in CA1, again suggesting a repulsion effect for three shared city trials. On the other hand, between-city PS for two shared city trials was not significantly lower than unique-city trials ($t(26) = 0.554$, $p = 0.584$, CI = [0.002 ± 0.006], Cohen's d = 0.107, two-tailed, FDR corrected) but was significantly higher than three city shared trials between-city PS ($t(26) = 3.012$, $p = 0.011$, CI = [0.006 ± 0.005], Cohen's d = 0.580, two-tailed, FDR corrected) in CA1, suggesting that the memory representation of two shared city trials did not show repulsion effect. We did not find, however, significant differences between unique-city trial PS and two ($t(26) = -0.516$, $p = 0.610$, CI = [-0.002 ± 0.006], Cohen's d = -0.009, two-tailed) or three shared city trials ($t(26) = 0.815$, $p = 0.422$, CI = [0.002 ± 0.005], Cohen's d = 0.157, two-tailed, FDR corrected) between-city PS in CA2/3/DG (Supplementary Figure 5). When a relative metric of representations as a new index of the repulsion effect was calculated, the result again revealed a repulsion effect in CA1 ($t(26) = -3.349$, $p = 0.004$, CI = [-0.008 ± -0.005], Cohen's d = -0.671, compared to zero, two-tailed), but not in CA2/3/DG ($t(26) = -0.851$, $p = 0.422$, CI = [-0.002 ± -0.005], Cohen's d = -0.157, compared to zero, two-tailed). These findings mirror the analyses reported in the results section and suggest that the number of trials in each comparison was not a confounding factor in our analyses.

Supplementary Note 6 (related to Supplementary Figure 6): addressing possible confounds in the leave-one-city-out SVR classification. There were common stores shared across the three cities (see Fig.1b); thus, it was possible that the leave-one-city-out cross-validation SVR classification result might be driven by these stores shared across cities. We calculated the count of shared trials across cities in each leave-one-city-out classification iteration. For example, when City 1 and City 2 were used as the training set, and City 3 as the test set, we could calculate how many trials of City 1 and 2 shared with City 3. Supplementary Figure 5c showed the number of shared trails in each iteration. City 2 had the highest number of shared trials with City 1 and 3, while City 3 had the lowest number of shared trials with City 1 and City 2 (City 1 and City 2: $t(26) = -9.578$, $p < 0.001$, CI = [-3.519 ± 0.756], Cohen's d = -1.843; City 2 and City 3: $t(26) = 20.315$, $p < 0.001$, CI = [9.222 ± 0.933], Cohen's d = 3.910; City 1 and City 3: $t(26) = -11.339$, $p < 0.001$, CI = [5.704 ± 1.034], Cohen's d = 2.182; Supplementary Figure 5c). The more trials shared between training and test sets indicated a greater possibility of generalization and a higher classification accuracy in this iteration. However, after we examined the classification accuracy for each city, we did not find a significant difference across the three cities ($t(26) < 1.138$, *Ps* > 0.266, Cohen's ds < 0.219, Fig.4b and d). This control analysis ruled out that the leave-one-city out classification results were driven by the shared stores across cities.

Supplementary Note 7 (related to Supplementary Figure 7): simulation. The simulation explores the question of whether distributed changes in either single neural firing or local field potentials (LFP) might reflect at the level of summed activity with BOLD using MVPA (the methods employed in this paper). Note this is a different question than whether univariate increases or decreases in neural activity relate to BOLD and instead focuses on distributed patterns across a brain structure. Also, please note that the simulations do not assume a topography to hippocampal neural responses and simulate these as random[3].

Imagine that each matrix above represents a collection of randomly distributed hippocampal neurons active in some form during exploration of four different environments (Supplementary Figure 7a). Matrix 1&2 represent two different environments, which are different a manifest as distinct neural patterns of activity ($r = -.001$). These patterns could be thought of as representing environments 1 and 2. Then, we construct two new matrices, matrix 3 and 4, by randomly placing 1s such that matrix 3 is positively correlated with matrix 2 ($r = 0.582$) but matrix 4 is negatively correlated with matrix 3 ($r = -0.335$). The idea here is that matrix 3 is an environment related in some form to that explored in matrix 2. Matrix 4 represents the situation of “repulsed” representations we have explored in the main sections of the manuscript. The correlations are shown in a bar graph (Supplementary Figure 7b).

We could imagine matrix 2&3 representing two different environments that have some commonalities (perhaps even the same environment but on different sessions) while matrix 3&4 share interfering characteristics and thus show hypothesized repulsion (a below zero correlation in this case but anything below "orthogonal" works). While the latter (repulsed representation) have never been shown with single neuron or LFP recordings, they have been shown with fMRI [4].

Therefore, we asked the question whether binning and summing the matrices above would produce similar correlation structures as the matrices shown above? The idea is that the local field potential or single neurons, when appropriately summed across all neurons/areas, would produce some measurable signal in BOLD. While we agree this assumption must be tested empirically, the idea here is to determine *whether* it is reasonable at all to hypothesize a correspondence between pattern completion/separation/repulsion at the single neuron/LFP level and fMRI MVPA based on distributed coding mechanisms.

Supplementary Figure 7c shows what the new binned matrices (with voxels 1/10th the original resolution) look like. Binned matrix 1 and matrix 2 continue to show little correlation (r = -.008) while binned matrix 3 continues to show a positive correlation with matrix 2 (r = 0.594) and binned matrix 4 continues to show a negative correlation with matrix 3 (r = -0.257).

This is again shown in a bar graph (Supplementary Figure 7d). In other words, the binning and summing does not destroy the basic structure of the correlation plots shown with the original matrices.

To ensure that this was somehow not a property of our particular randomly selected values, we conducted the simulation 1000 times with new random values and matrices. The plot shows the similarity (correlation) between the original and binned matrix correlations (Supplementary Figure 7e). Notice the histogram is strongly skewed to the right, suggesting that the results are stable across different randomizations.

We ran the simulations above by randomly replacing ones at locations in matrix 3 and 4 to produce positive and negative correlations. To assure that this was not an artifact of our procedure, we ran the simulations instead using zeros (Supplementary Figure 7f) or random numbers (Supplementary Figure 7g). We obtained identical results to before, showing that the correlation structure is preserved when binning and summing even with different matrix configurations.

To be clear, these simulations do not show that there must be a correspondence between pattern completion / separation at the neural level and that at the voxel level. In fact, if the assumption of summation of activity, either with the LFP or single neurons, does not correspond to some distributed observable change in the BOLD signal, we would not see any relationship between these mechanisms and levels. At the same time, given that neural activity is at least one component of the BOLD signal, it seems

reasonable to assume that at the level of *distributed activity*, there might be some correspondence here. Note that previous experiments that have shown little correlation between the BOLD signal and LFP / single neuron changes have looked at univariate changes. In other words, these studies have shown no relationship between increases in either single neurons or the LFP as they relate to increases in BOLD[5,6]. In contrast, our simulation asks whether distributed patterns, particularly changes in distributed patterns of either single neuron or LFP activity, could be reflected in the BOLD signal. This question has not been answered experimentally and remains to be addressed. Importantly, however, these simulations suggest that voxels that represent summations of either neural or LFP changes between environments could show similar changes at the level of the BOLD signal. Note that studies that have looked at this issue in other brain structures have shown similar correspondences between multivariate patterns at the level of fMRI and single neurons / LFPs[7,8]. In other words, pattern completion / separation / and repulsion all could *hypothetically* arise at the distributed level in the BOLD signal from either the single neuron or LFP activity.

Supplementary References

1 Mikolov, T., Chen, K., Corrado, G. & Dean, J. Efficient estimation of word representations in vector space. *arXiv preprint arXiv:1301.3781* (2013).

2 Dimsdale-Zucker, H. R. & Ranganath, C. in *Handbook of behavioral neuroscience* Vol. 28 509-525 (Elsevier, 2018).

3 Redish, A. D. *et al.* Independence of firing correlates of anatomically proximate hippocampal pyramidal cells. *J Neurosci* **21**, RC134 (2001).

4 Favila, S. E., Chanales, A. J. & Kuhl, B. A. Experience-dependent hippocampal pattern differentiation prevents interference during subsequent learning. *Nat Commun* **7**, 11066, doi:10.1038/ncomms11066 (2016).

5 Ekstrom, A., Suthana, N., Millett, D., Fried, I. & Bookheimer, S. Correlation Between BOLD fMRI and Theta-Band Local Field Potentials in the Human Hippocampal Area. *Journal of Neurophysiology* **101**, 2668-2678, doi:10.1152/jn.91252.2008 (2009).

6 Hill, P. F. *et al.* Distinct neurophysiological correlates of the fMRI BOLD signal in the hippocampus and neocortex. *Journal of Neuroscience* (2021).

7 Dubois, J., de Berker, A. O. & Tsao, D. Y. Single-unit recordings in the macaque face patch system reveal limitations of fMRI MVPA. *Journal of Neuroscience* **35**, 2791-2802 (2015).

8 Kriegeskorte, N. *et al.* Matching categorical object representations in inferior temporal cortex of man and monkey. *Neuron* **60**, 1126-1141 (2008).
