# Supplementary material for: Partially overlapping spatial environments trigger reinstatement in hippocampus and schema representations in prefrontal cortex
Source: Nat Commun. 2021 Oct 28;12:6231. doi: 10.1038/s41467-021-26560-w (PMC8553856; doi:10.1038/s41467-021-26560-w)
Supplement: Supplementary file 3 — Reporting summary [file 41467_2021_26560_MOESM3_ESM.pdf]

nature research

Corresponding author(s): Arne D. Ekstrom
Last updated by author(s): 09/21/2021

Reporting Summary

Nature Research wishes to improve the reproducibility of the work that we publish. This form provides structure for consistency and transparency in reporting. For further information on Nature Research policies, see our Editorial Policies and the Editorial Policy Checklist.

Statistics

For all statistical analyses, confirm that the following items are present in the figure legend, table legend, main text, or Methods section.

n/a	Confirmed	
☐	☒	The exact sample size (n) for each experimental group/condition, given as a discrete number and unit of measurement
☐	☒	A statement on whether measurements were taken from distinct samples or whether the same sample was measured repeatedly
☐	☒	The statistical test(s) used AND whether they are one- or two-sided<br>*Only common tests should be described solely by name; describe more complex techniques in the Methods section.*
☐	☒	A description of all covariates tested
☐	☒	A description of any assumptions or corrections, such as tests of normality and adjustment for multiple comparisons
☐	☒	A full description of the statistical parameters including central tendency (e.g. means) or other basic estimates (e.g. regression coefficient) AND variation (e.g. standard deviation) or associated estimates of uncertainty (e.g. confidence intervals)
☐	☒	For null hypothesis testing, the test statistic (e.g. F, t, r) with confidence intervals, effect sizes, degrees of freedom and P value noted<br>*Give P values as exact values whenever suitable.*
☐	☒	For Bayesian analysis, information on the choice of priors and Markov chain Monte Carlo settings
☒	☐	For hierarchical and complex designs, identification of the appropriate level for tests and full reporting of outcomes
☐	☒	Estimates of effect sizes (e.g. Cohen's d, Pearson's r), indicating how they were calculated

Our web collection on statistics for biologists contains articles on many of the points above.

Software and code

Policy information about availability of computer code

Data collection	Unity3D version 2018.4.3 (https:// unity3d.com); PsychoPy3 (https://www.psychopy.org); Siemens 32-Channel 3T "Skyra" scanner in the University of Arizona.
Data analysis	MATLAB R2019b; classification analyses on the fMRI data were run using LIBSVM 3.12 implemented in MATLAB; FSL version 5.0.11; Feat version 6.0.0; ANTs 2.3.1; Automatic hippocampal subfield segmentation software(ASHS) which is built in ITK-SNAP (version 3.8.0); R version 4.0.2 (R Core Team, 2020); JASP 0.14.1.0; SPSS version 25.

For manuscripts utilizing custom algorithms or software that are central to the research but not yet described in published literature, software must be made available to editors and reviewers. We strongly encourage code deposition in a community repository (e.g. GitHub). See the Nature Research guidelines for submitting code & software for further information.

Data

Policy information about availability of data

All manuscripts must include a data availability statement. This statement should provide the following information, where applicable:

- Accession codes, unique identifiers, or web links for publicly available datasets
- A list of figures that have associated raw data
- A description of any restrictions on data availability

Data from the experiment is available on request form the corresponding author.

Field-specific reporting

Please select the one below that is the best fit for your research. If you are not sure, read the appropriate sections before making your selection.

- [x] Life sciences
- [ ] Behavioural & social sciences
- [ ] Ecological, evolutionary & environmental sciences

For a reference copy of the document with all sections, see nature.com/documents/nr-reporting-summary-flat.pdf

Life sciences study design

All studies must disclose on these points even when the disclosure is negative.

Sample size	A total of 32 right-handed participants were scanned in this study (27 included in the final sample). No statistical methods were used to predetermine sample sizes, but our sample size is similar to those reported in previous publications (Dimsdale-Zucker, et al., 2018, N = 23; Hindy et al., 2019, N = 24; Julian et al., 2021, N = 24).
Data exclusions	Four participants were excluded from the analysis due to excessive movement (greater than 1 voxel), and one participant was excluded due to an incidental finding.
Replication	We do not offer a replication, this is a single fMRI study. Future research should aim to replicate our findings.
Randomization	The experiment was designed as a within-participant study, thus all participants participated in all experimental conditions.
Blinding	Blinding was not relevant as this was a within-participant study. All participants were unaware of the manipulation.

Reporting for specific materials, systems and methods

We require information from authors about some types of materials, experimental systems and methods used in many studies. Here, indicate whether each material, system or method listed is relevant to your study. If you are not sure if a list item applies to your research, read the appropriate section before selecting a response.

Materials & experimental systems

n/a	Involved in the study	
x		Antibodies
x		Eukaryotic cell lines
x		Palaeontology and archaeology
x		Animals and other organisms
	x	Human research participants
x		Clinical data
x		Dual use research of concern

Methods

n/a	Involved in the study	
x		ChIP-seq
x		Flow cytometry
	x	MRI-based neuroimaging

Human research participants

Policy information about studies involving human research participants

Population characteristics	A total of 32 right-handed participants were recruited from the Tucson community and were compensated for their time. Four participants were excluded from the analysis due to excessive movement (greater than 1 voxel), and one participant was excluded due to an incidental finding. Therefore, the final sample size was comprised of 27 participants (17 females, mean age: 22.52 years, range: 18-35 years). All participants had normal or normal-to-corrected vision and normal color perception. Based on self-report, all participants were screened to ensure they had no neurological conditions.
Recruitment	A total of 32 right-handed participants were recruited from the Tucson community. The recruitment of college students is common in neuroimaging studies and we do not foresee any specific bias in the results. All measures were taken within each individual participant.
Ethics oversight	The study was approved by the Institutional Review Board at the University of Arizona and written Informed consent was obtained from each participant prior to the experiment.

Note that full information on the approval of the study protocol must also be provided in the manuscript.

Magnetic resonance imaging

Experimental design

Design type	The study was an event-related design
Design specifications	Each subject completed 12 runs of memory retrieval runs and 2 runs of the localizer task. Each retrieval run contained 15 trials and each trial was 18.13s. Each localizer run contained 18 trials. Each trial was 18.13s.
Behavioral performance measures	Correct button responses and RT were recorded for retrieval trials and the localizer runs.

Acquisition

Imaging type(s)	functional and structural
Field strength	3 Tesla
Sequence & imaging parameters	High-resolution functional images were acquired using a simultaneous multi slice whole-brain echo planar imaging (EPI) sequence (interleaved acquisition, TR = 2590 ms, TE = 30 ms, flip angle = 82 degree, field of view (FOV) = 234 mm, matrix = 128 × 128, slice thickness =1.8 mm, slices = 84, slice acceleration factor = 3, phase encoding direction = right to left, bandwidth = 1562 Hz/pixel), adapted from a previous study80. High-resolution structural images were obtained using a 3D, T1-weighted MPRAGE (1 mm3 isotropic) sequence acquired for the whole brain (FOV = 256 mm, matrix = 256 × 256, slice thickness =1 mm, TR = 2100 ms, TE = 2.33 ms, flip angle = 12 degree, bandwidth = 190 Hz/pixel). High-resolution anatomical images of the hippocampus and surrounding cortex were acquired with a T2-weighted turbo-spin echo (TSE) anatomical sequence (FOV = 200 mm × 200 mm, matrix = 448 × 448, TR = 4200.0 ms, TE = 93.0 ms, flip angle = 139 degree, slice thickness = 1.8 mm, 28 slices, bandwidth = 199Hz/pixel). Sequences were acquired perpendicular to the long axis of the hippocampus. An additional coplanar matched-bandwidth high-resolution gradient echo EPI sequence (TR = 6120 ms, TE = 39 ms, slices = 84, FOV = 245 mm, matrix = 128 × 128, flip angle = 90 degree, bandwidth = 1446 Hz/pixel) was acquired to aid in registration of the EPI sequence to the high-resolution structural images. B0-field maps were acquired immediately with a gradient recalled echo sequence (TR = 888.0 ms, TE1 = 4.92 ms,TE2 = 7.38 ms, flip angle = 90 degree, FOV = 256 mm, slice thickness =3 mm, slices = 84) following the coplanar matched-bandwidth sequence to correct for inhomogeneities of the magnetic field81. This sequence covered the whole brain, allowing us to correct field distortions for the entire EPI sequence.
Area of acquisition	whole brain for T1-MPAGE and EPI;<br>a partial volume including the hippocampus and surrounding cortex acquired with a T2-weighted turbo-spin echo (TSE) anatomical sequence.

Diffusion MRI ☐ Used ☒ Not used

Preprocessing

Preprocessing software	Image preprocessing was performed by using FEAT (FMRI Expert Analysis Tool), version 6.00, implemented in FSL (part of the FSL package; http://www.fmrib.ox.ac.uk/fsl).
Normalization	All functional images were linearly registered to individual-subject T1 MPRAGE structural volumes in a two-step process via a coplanar matched-bandwidth sequence described above using FLIRT. Registration from structural images to the standard MNI-152 template was further refined using FNIRT nonlinear registration. Statistical analyses were performed in the image space, with the statistical maps normalized to the standard space (FSL's MNI 152 template) for higher level group analysis in when needed.
Normalization template	MNI 152 template.
Noise and artifact removal	The EPI images were first corrected for geometric distortion using participants' field maps and underwent motion-correction. Six motion parameters were added as confound variables to the model. Residual outlier timepoints were identified using FSL's motion outlier detection program and integrated as additional confound variables in the first-level general linear model (GLM) analysis.
Volume censoring	We censored volumes using a framewise displacement (FD) threshold of 0.5 mm and excluded excluded any trials with any censored frames during the duration of the modeled GLM response using a FD threshold of 0.5 mm.

Statistical modeling & inference

Model type and settings	The GLMs were performed separately to estimate the activation pattern for each of 180 retrieval trials and 36 localizer trials. In this single-trial model, a Least Square – Separate (LS-S) approach was used, in which the trial of interest was modeled as one regressor, with all other trials modeled as a separate regressor. The t-map for each trial was used for multivariate pattern analysis (MPS) and classification analysis.<br>MPS: Average similarity was computed per participant and condition and entered to group level statistics.<br>Classification analysis: accuracy was computed for each participant and entered to group level statistics.
Effect(s) tested	MPS: a three-way repeated-measure ANOVA, with the factors of 6 ROIs of MTL, 8 t-stat thresholds (tSNR) levels, and three conditions (unique store within-city PS / shared store within-city PS / unique store between-city PS) as within-subject

variables was conducted. Another two-way repeated measure ANOVA, with the factors of 8 TSNR levels and three conditions (between-city PS for unique city trials, between-city PS for two shared city trials, and between-city PS for three shared city trials) as within-subjects variables was conducted to examine repulsed representations. T-test were used to test pairwise simple effects between conditions.
Searchlight leave-one-city out classification analysis: The resulting correlation coefficients were transformed into Fisher's z-scores and then input into further group analyses using a cluster-forming threshold of $Z > 3.1$, with $p < 0.05$ (corrected for family-wise error rate, using random field theory).
ROI based leave-one-city-out classification analysis: T-test, compared with chance level (i.e., zero)
Searchlight-based MPS: The resulting correlation coefficients were transformed into Fisher's z-scores and then input into further group analyses using a cluster-forming threshold of $Z > 2.6$, with $p < 0.05$ (corrected for family-wise error rate, using random field theory).

Specify type of analysis: ☐ Whole brain ☐ ROI-based ☒ Both

Anatomical location(s): Automatic hippocampal subfield segmentation software (ASHS) was used to segment the subregions of the MTL based on each participant's high-resolution T2-weighted MRI image. The MTL was segmented into CA1, CA2/3, DG, and subiculum (SUB), perirhinal cortex (PRC) and entorhinal cortex (ERC) and parahippocampus cortex (PHC). We combined the CA2/3 and DG subfields as finer distinctions cannot be made at the acquired resolution. Single-trial t-map were then obtained within those 6 ROIs (CA1, CA2/3/DG, SUB, ERC, PRC, PHC) for each subject for further MPS and classification analysis. Following a previous study, the medial PFC was defined as a set of three regions within the Brodmann areas (BA) 10, 11 and 32.

Statistic type for inference
(See Eklund et al. 2016)

Searchlight based analysis: The resulting correlation coefficients were transformed into Fisher's z-scores and then input into further group analyses using a cluster-forming threshold of $Z > 3.1$, with $p < 0.05$ (corrected for family-wise error rate, using random field theory).
Correlating Frontal Activity with hippocampal PS: group images were thresholded using cluster detection statistics, with a height threshold of $Z > 2.3$ and a cluster probability of $P < 0.05$, corrected for whole-brain multiple comparisons using Gaussian Random Field Theory.

Correction: FWE (family-wise error, $p < 0.05$) for voxel-wise inference and FDR correction for all ROI analysis

Models & analysis

n/a	Involved in the study
☒	☐ Functional and/or effective connectivity
☒	☐ Graph analysis
☐	☒ Multivariate modeling or predictive analysis

Multivariate modeling and predictive analysis

Single-trial response estimates
The GLMs were performed separately to estimate the activation pattern for each of 180 retrieval trials and 36 localizer trials. In this single-trial model, a Least Square – Separate (LS-S) approach was used, in which the trial of interest was modeled as one regressor, with all other trials modeled as a separate regressor88. Specifically, each single-trial GLM included 5 regressors: (1) the trial of interest; (2) all other trials; (3) black outline stage; (4) fixation; (5) all incorrect trials within the active baseline task. Each event was modeled at the time of stimulus onset and convolved with a canonical hemodynamic response function (double gamma), whereas the correct baseline trials (X/O judgement task) were not coded and thus were treated as an implicit baseline. To control for the effects of head motion, six-motion parameters were included in the GLM model as a covariate. The t-map for each trial was used for multivariate pattern analysis (MPS) and SVR classification analysis to increase the reliability by normalizing for noise.

Run-based response estimates for SVM classification analysis
The GLMs were performed separately to estimate the activation pattern for each retrieval run. Each single-run GLM included 6 regressors: (1) the remembered trials; (2) forgotten trials; (3) missed trials; (4) black outline stage; (5) fixation; (6) all incorrect trials within the active baseline task. Each event was modeled at the time of stimulus onset and convolved with a canonical hemodynamic response function (double gamma), whereas the correct baseline trials (X/O judgement task) were not coded and thus were treated as an implicit baseline. To control for the effects of head motion, six-motion parameters were included in the GLM model as a covariate. This resulted in 4 run-based data points per city per subject. The run-based t-map has greater reliability and could be used for SVM classification analysis to increase accuracy and power.
